# The impact of cognitive load on operatic singers' timing performance

**DOI:** 10.3389/fpsyg.2015.00429

**Published:** 2015-04-22

**Authors:** Muzaffer Çorlu, Pieter-Jan Maes, Chris Muller, Katty Kochman, Marc Leman

**Affiliations:** Department of Musicology, Institute for Psychoacoustics and Electronic Music, Ghent UniversityGhent, Belgium

**Keywords:** dual task, cognitive load, working memory, operatic singing performance, timing

## Abstract

In the present paper, we report the results of an empirical study on the effects of cognitive load on operatic singing. The main aim of the study was to investigate to what extent a working memory task affected the timing of operatic singers' performance. Thereby, we focused on singers' tendency to speed up, or slow down their performance of musical phrases and pauses. Twelve professional operatic singers were asked to perform an operatic aria three times; once without an additional working memory task, once with a concurrent working memory task (counting shapes on a computer screen), and once with a relatively more difficult working memory task (more shapes to be counted appearing one after another). The results show that, in general, singers speeded up their performance under heightened cognitive load. Interestingly, this effect was more pronounced in pauses—more in particular longer pauses—compared to musical phrases. We discuss the role of sensorimotor control and feedback processes in musical timing to explain these findings.

## Introduction

Expressive music performance requires a fine-grained temporal coordination of muscle activity to control one's musical instrument, or vocal chords in the case of singing performance. Thereby, musicians often perform under conditions of heightened cognitive load due to various reasons. Previous research demonstrated that a cognitive load impairs regular timing production, suggesting the role of a cognitively controlled system for the temporal control of body movements (Krampe et al., 2010; Rattat, 2010; Fischinger, 2011; Çorlu et al., 2014; Maes et al., 2014). The basic idea is that a dedicated internal clock is used to keep track of time. The most influential account of this “timekeeper” approach is the pacemaker-accumulator model (Gibbon, 1977). In this model, a clock, or *pacemaker* emits pulses that enter an *accumulator* via an attention-controlled switch. The number of accumulated pulse is stored in working memory, and compared with a criterion interval in reference memory. Baddeley (1966) described working memory as a process by which information is stored and processed. A typical effect that is observed in experiments investigating timing production under heightened cognitive load is a tendency to speed up (Krampe et al., 2010; Rattat, 2010; Çorlu et al., 2014; Maes et al., 2014). This effect is explained by memory-base models of estimated time duration, such as Ornstein's storage-size hypothesis that states that the experience of duration is related to the amount of stored information: as the storage size increases, duration experience increases (Ornstein, 1969). Accordingly, in situations of heightened cognitive load, cognitive storage size will increase more rapidly, leading to an overestimation of interval durations, and correspondingly to the production of shorter temporal intervals. This cognitive timekeeper approach is highly vulnerable to cognitive load and therefore relatively inefficient in situations that require heightened cognitive load. Accordingly, this approach is presumably incomplete to fully explain timing behavior. Current research suggests that perceptual and motor systems may guide the temporal control of body movements in interaction with the external environment (Jones and Boltz, 1989; Hopson, 2003; Mauk and Buonomano, 2004; Ross and Balasubramaniam, 2014). In the course of coordinating body movements, (repeated) patterns in spatial trajectory and energy expenditure (e.g., muscle contractions/relaxations) arise that can be used to “index” time in a continuous way. Accordingly, it is suggested that temporal control may emerge from the control of movement dynamics itself, without the need for a central timekeeper. This “emergent” timing approach is supported by research investigating the mechanisms underlying the temporal control of continuous and discrete rhythmic movements, defining respectively an *emergent* and *event* timing system (Robertson et al., 1999; Zelaznik et al., 2002, 2005, 2008; Delignières et al., 2004; LaRue, 2005; Torre and Balasubramaniam, 2009; Studenka et al., 2012). Other research, focusing on the role of sensory information in timing production tasks, suggested that sensory information coming from the external environment, as well as self-generated sensory feedback may contain temporal cues that guide temporal behavior in a more or less direct way (Rodger and Craig, 2011; Varlet et al., 2012; Roerdink et al., 2013; Bravi et al., 2014). By repeated experience, and general (associative) learning mechanisms, people learn how patterns of dynamic change in sensory information provide an index of the passage of time (Dragoi et al., 2003; Hopson, 2003; Addyman et al., 2011). Correspondingly, proper timing may then be realized by “anchoring” muscle activation to these sensory patterns.

In line with this body of research, we claim that people can rely on self-generated sensory feedback and sensorimotor control to regulate temporal coordination of muscle activity. In the current study, we tested this hypothesis in the context of expressive music performance, more in particular singing performance. A singing performance is a naturalistic task that requires auditory-motor coordination and expressive timing control, often under conditions of heightened cognitive load. We used operatic singers, as they often encounter situations, in which they have to perform under heightened cognitive load; they have to act and know where to move around on the stage, to know when to sing something, and to interact with others. Also, singers have the added charge of language, which involves correct pronunciation, natural inflection, clear diction, and genuine comprehension in as many as four different languages beside one's own (Helding, 2012). According to Kleber et al. (2010), operatic singers develop specialized neural networks for enhanced somatosensory processing and performance monitoring, as well as motor sequence attention when compared to laymen and even other singers. The findings suggest that changes in the primary sensorimotor cortex (S1), inferior parietal lobule (IPL), and dorsolateral prefrontal cortex (DLPFC) allow for more accurate fine tuning and feed forward motor commands. Besides, with singers it is easier to define pause areas than with other instrumentalists. In vocal performance respiratory behavior is rule based. Singers should only breathe at predefined areas between phrases, rests, and punctuation.

For the purpose of the study, we applied a dual-task interference paradigm (Pashler, 1994). This paradigm assumes that when two tasks rely on similar processing resources at the same point in time, interference will occur due to the inherent limitations of the processing resources. This approach allows pinpointing the role of cognitive and sensorimotor resources in the temporal control of a singer's voice in an expressive performance. The primary task consists of singing an operatic aria, and the secondary task is a working memory task, in which participants count the number of shapes appearing on a computer screen.

The main aim of the study was to investigate to what extent the additional working memory task (cognitive load) affected expressive timing of the singers' performance. Thereby, we focused on performers' tendency to speed up, or slow down. The operatic arias that the participants of our study sang contained both musical phrases and pauses. We expected that, in general, musical timing would be affected by the concurrent working memory task (cognitive load). More in particular, we expected a tendency of singers to speed up their performance. However, based on the above-mentioned theories, we hypothesized that phrases and pauses would be affected differently by the working memory task.

**Figure 1:**
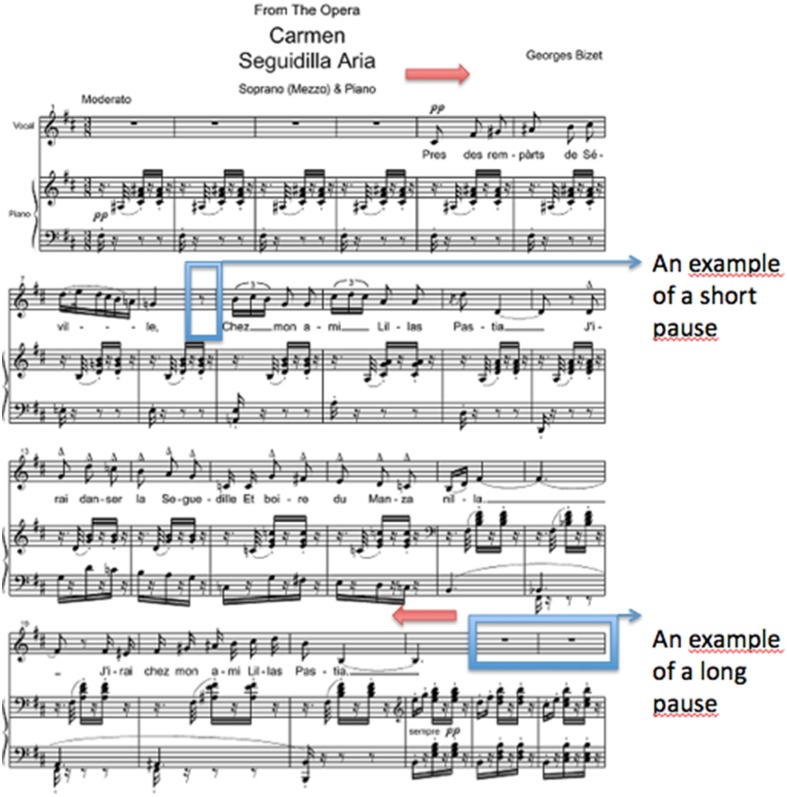
**The entrance passage of The Carmen Opera by Georg Bizet**. An example of a short and a long pauses are indicated. A musical phrase is in between red arrows.

By phrase we mean; musical sentences that involve a series of notes, and by pauses we mean the silent regions that usually occur between musical phrases or sometimes within the phrases (see Figure 1). On the one hand, there are pauses in between phrases that are not only for breathing, in terms of musical structure; those are also part of the score. Therefore, these pauses are long enough to breath and to count for the next phrase (usually a whole rest or double whole rest). But on the other hand, there are shorter pauses where musicians can only breathe that are usually within the phrases (usually an eight rest or sixteenth rest).

During pauses—in particular longer pauses that are not used solely for breathing—we expected singers to rely on cognitive timekeeper resources, as there is no sensory and sensorimotor feedback to rely on. In contrast during phrases, sensory feedback and sensorimotor control may function as a “scaffold” in support of temporal control of the singing voice. Also, respiration in shorter pauses could possibly function as somatosensory scaffold in support of musical timing. In conclusion, we expected that increases in cognitive load would affect longer pauses more, compared to phrases and shorter pauses used solely for breathing, because timing at these points in time rely on the same processing resources, as does the cognitive task performance.

## Methods

### Participants

Twelve operatic singers (six female, mean age = 29.2 years; range = 29–33 years) participated in the experiment. All participants were right-handed. They had at least 8 years of formal musical training, and at least 8 years of stage experience. All were Turkish native speakers (also Turkish maternal speech). Written informed consent was obtained from all participants prior to participation, and the Ethical Review Committee of Ghent University reviewed the experiment.

### Stimuli

#### Operatic arias

For the primary musical task, the operatic singers were asked to sing an operatic aria from their repertoire (see Table 1). The arias were at least 100 s in duration. Males sang arias for males and females sang arias for female. The arias were selected based on the repertoire with which they were acquainted and felt comfortable singing. Two sopranos chose the aria “Voi che sapete” from Le Nozze di Figaro by Mozart (Table 1).

**Table 1:** **Operatic arias chosen by the singers**.

**Composer**	**Opera, role**	**Aria**
G. Bizet	Les pécheurs de perles, Leila	“Comme autrefois”
G. Bizet	Carmen, Carmen	“Seguidilla”
G. Bizet	Carmen, Don jose	“La fleur que tu m'avais jetée sospiro”
G. Verdi	La traviata, Alfredo	“Lunge da lei/ De'miei bollenti spriti”
W. A. Mozart	Le Nozze di Figaro, Count Almaviva,	“Vedro mentr'io”
W. A. Mozart	La Clamenza di Titto, Sextus	“Parto Parto”
W. A. Mozart	Le Nozze Di Figaro, Cherubino	“Voi che sapete”
W. A. Mozart	Don Giovanni, Leporello	“Madamina il catalogo e questo”
Charles Gounod	Faust, Valentin	“Avant de quitter ces lieux”
Jules Massenet	Werther, Charlotte	“Va ! laisse couler mes larmes”

Classical singers are governed strictly by a fach system to assist in the categorization of instruments (light or dramatic, range, etc.) This is important due to the physical parameters and capabilities of the voice. As it was necessary to avoid confounds resulting from imposed repertoire, they were allowed to sing the material that was typical for their voice. Later, studies can compare singers using the same repertoire, but it was important for this study design that it be as ecological as possible. The individual compositional features of the music will always have an impact in these cases, but an overall statistical trend may still be initially identified.

#### Working memory task

For the secondary working memory task, different shapes (squares, circles, and triangles) were presented for 800 ms each, until a next shape appeared. In condition one (no additional load condition) a 100 s movie was displayed in which blue squares appeared on the screen at random locations, one square at a time. In condition two (working memory task condition with low load), again a 100 s movie was displayed where squares, circles and triangles randomly appeared. The exact number of triangles was seven and the exact number of squares was eight. Condition three (working memory task condition with high load) was the same as condition two, only the number of shapes differed: 12 triangles and 16 squares had to be counted.

In no load condition participants were asked to look at the visual objects, but not to count anything. In all conditions, singers were observed as they were looking at the computer screen.

### Materials and apparatus

Participants were placed at approximately 1 m from a 15-inch flat screen computer monitor. The experiment took place in a professional sound recording studio. The entire procedure was automated using a computer patch programmed in Max/MSP (http://cycling74.com/products/max/), which ran on a MacBook Pro. The patch displayed written instructions for the participants to start and stop playing, it played the movies of the working memory task, and it automatically handled synchronized recordings of the audio. During performances, video footage (using a Canon Legria digital camera) was recorded.

### Design and procedure

Upon arrival, participants were given a short explanation about the experiment, they read the information sheet that explained the whole procedure, and they signed the consent form. The experimental procedure was organized in three different conditions (no/low/high cognitive load) that were counterbalanced across participants. Participants performed each condition once. Between conditions, participants were given a short resting period of approximately 1 min.

No metronome was used. Singers were instructed to sing their pieces exactly the same way three times. Counterbalanced experimental order was used to eliminate the possibility that tempo changes, due to the experimental manipulations, had not occurred randomly.

At the beginning of each condition, the word “start” appeared on the screen indicating to the participants to start singing, while the 100 s movie (see Section Stimuli) started concurrently. Participants were instructed to sing their aria in exactly the same way in each condition. In the no load condition, participants were asked to sing while only looking at squares appearing on the computer screen. In the experimental conditions (low/high load), participants were asked to sing while concurrently counting the number of circles and the number of triangles appearing on the screen. So, participants had to store and manipulate two separate numbers in memory. After 100 s, the word “stop” appeared on the screen indicating to the participant to stop singing. The total singing duration was measured as follows: the starting point was always when a singer starts to sing (visually determined based on the waveform). Some singers took some time before starting their performance, which accounted for the fact that the total duration of their performance was less than 100 s. Afterwards, participants were asked to report the number of circles and the number of triangles they counted. In between conditions there were 2 min of resting period. In all conditions, singers were observed as they were looking at the computer screen. In order not to interfere, the observer sat a bit behind the singers.

The participants filled out a brief questionnaire regarding their personal and musical background after they performed in all three conditions.

### Audio analysis

**Figure 2:**
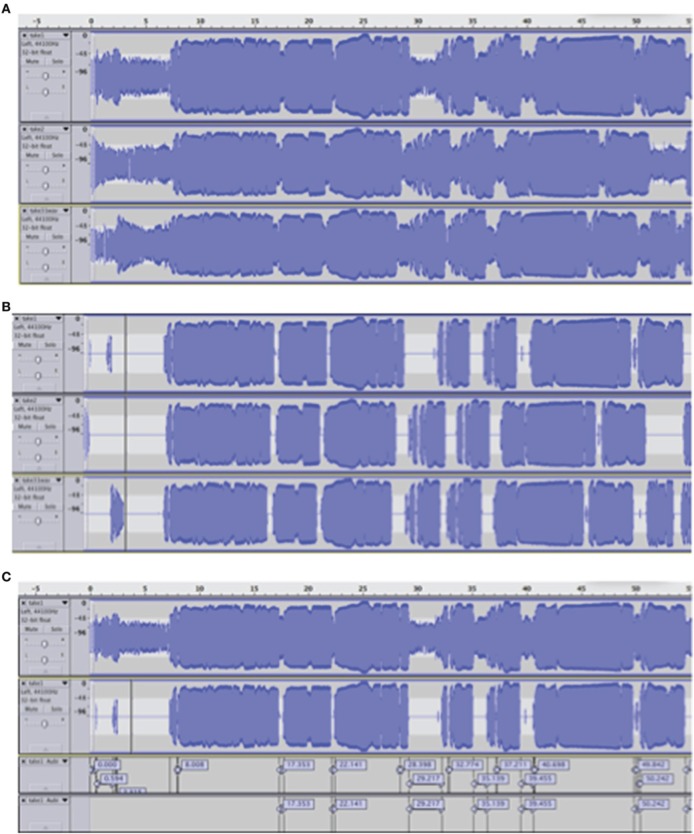
**(A)** Raw audio data sample of one singer: no load condition 1 (top), low load condition (middle), high load condition (bottom). **(B)** Audio track samples after the noise below −40 dB was removed from the three signals of Figure 2A. **(C)** An example of raw audio (the first layer corresponds with first layer of Figure 2A) and after noise removal (the second layer corresponds with the first layer of Figure 2B), with the silent regions extracted (the third layer is based on the data of the second layer). Finally, the fourth layer shows the silent regions that are taken into account after a manual inspection of the analysis.

Raw audio data (recorded performances) were imported into Audacity (http://audacity.sourceforge.net/). For each singer, the three audio tracks (one for each condition, Figure 2A) were displayed and extraneous noise was removed below −40 dB (Figure 2B), using the noise removal function of Audacity. The silent regions detector of Audacity was applied to segment the recorded performances into musical phrases and pauses, as a basis for further manual analysis (Figure 2C). In addition, changes in expressivity were taken into account.

For example, if a musician sang legato in one performance but non-legato in the other performance, pauses between non-legato notes were not considered silences. Based on the obtained audio performances, we calculated two dependent variables.

First, we were interested in changes in the overall duration of the performance in each condition (no/low/high load). In the no load baseline condition, singers stopped after 100 s. After inspection, it was found that the baseline condition for each participant was always performed at the slowest tempo compared to the low and high load conditions. Then we identified the positions in the audio recordings of the experimental conditions that corresponded with the position in the audio recording of the baseline condition after 100 s. Accordingly, we obtained three values for each participant that represented the total duration of their performances in each condition.

Second, we wanted to investigate whether the durations of musical phrases and pauses (i.e., Performance type) were differently affected by the working memory task. For that purpose, we calculated the total accumulated durations of both the musical phrases and pauses in the baseline condition. For each participant, these two values were taken as reference to compare the durations of musical phrases and pauses in the experimental conditions (low/high load). Accordingly, we calculated for each experimental condition the difference in duration in reference to the baseline, expressed as a percentage. Additionally, to further investigate whether effects of cognitive load were influenced by pause duration, we divided pauses into two categories (long/short) using a split-median analysis for each participant.

## Results

All effects are reported as significant at an alpha level of 0.05. *Post-hoc* tests for interactions were conducted with alpha levels corrected for multiple comparisons using Bonferroni's method. For the repeated-measure ANOVA tests, we tested for the assumption of sphericity using Mauchly's test. When the assumption of sphericity was violated, we corrected degrees of freedom using the Greenhouse-Geisser procedure.

### Total duration (s)

**Figure 3:**
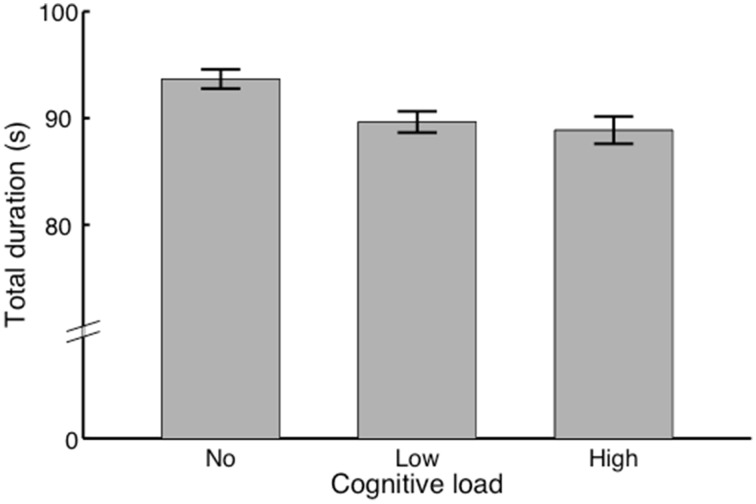
**Total singing durations of each condition**. Bars indicate the total durations of the performances in each condition in seconds. In other words how performances speed up with conditions.

We performed a repeated-measures ANOVA with Condition as within-subjects factor (no/low/high load). The results showed (see Figure 3) a significant main effect, *F*_(1, 11)_ = 10.08, *p* < 0.01, η^2^*_p_* = 0.48. *Post-hoc* comparisons yielded a significant difference between the no load condition (*M* = 93.67, SEM = 0.90) and low load condition (*M* = 89.64, SED = 0.99), *t*_(11)_ = 5.11, *p* < 0.001. Also, we found a significant difference between the no load condition (*M* = 93.67, SEM = 0.90) and the high load condition (*M* = 88.88, SEM = 1.28), *t*_(11)_ = 3.25, *p* < 0.05. No significant difference was found between the low load condition and the high load condition.

### Working memory task

In low load condition singers had to count seven triangles and eight circles. Here they counted (±2) and in high load condition, 12 triangles and 16 circles had to be counted. Singers counted roughly (±5).

### Duration (s) of musical phrases and pauses

**Figure 4:**
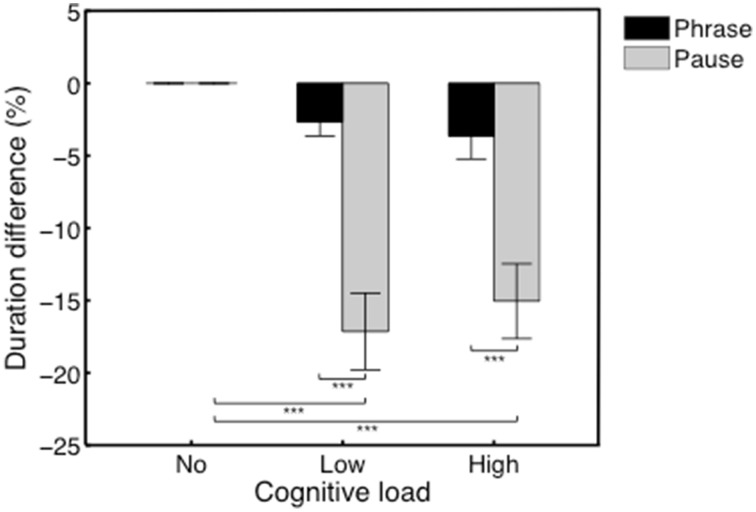
**Average duration difference (in %) of the phrases in the no/low/high load conditions, and of the pauses in the no/low/high load conditions**. A significant interaction effect was found driven by a significant decrease of the duration of pauses in the low and high load conditions. ^***^p ≤ 0.001.

Distributions of pause durations for each participant are shown in Figure 6. Average duration difference (in %) of the phrases in the no/low/high load conditions, and of the pauses in the no/low/high load conditions are shown in Figure 4. We performed a Two-Way repeated-measures ANOVA with Performance type (Phrase/Pause) and Condition (no/low/high load) as within-subjects factors. The analysis yielded a significant main effect for the factor Performance type, *F*_(1, 11)_ = 48.61, *p* < 0.001, η^2^*_p_* = 0.81, with Phrases (*M* = −2.10, SEM = 0.78) having a significantly different difference in percentage (the dependent variable) compared to Pauses (*M* = −10.73, SEM = 1.37). Also, we found a significant main effect of Condition, *F*_(2, 22)_ = 25.66, *p* < 0.001, η^2^*_p_* = 0.70. *Post-hoc* comparisons revealed significant differences between the no load condition and the low load condition (*M* = −9.90, SEM = 1.38), *t*_(11)_ = 7.09, *p* < 0.001, and between the no load condition and the high load condition (*M* = −9.34, SEM = 1.75), *t*_(11)_ = 5.36, *p* = 0.001. Additionally, a significant interaction effect was found between Performance type and Condition, *F*_(2, 22)_ = 11.86, *p* < 0.001, η^2^*_p_* = 0.52. *Post-hoc* comparisons indicated that the interaction was driven by the significant decrease of the duration of pauses in the low load condition (*M* = −17.15, SEM = 2.65), *t*_(11)_ = 6.47, *p* < 0.001, and high load condition (*M* = −15.05, SEM = 2.59), *t*_(11)_ = 5.82, *p* < 0.001.

### Effects of pause duration (s)

**Figure 5:**
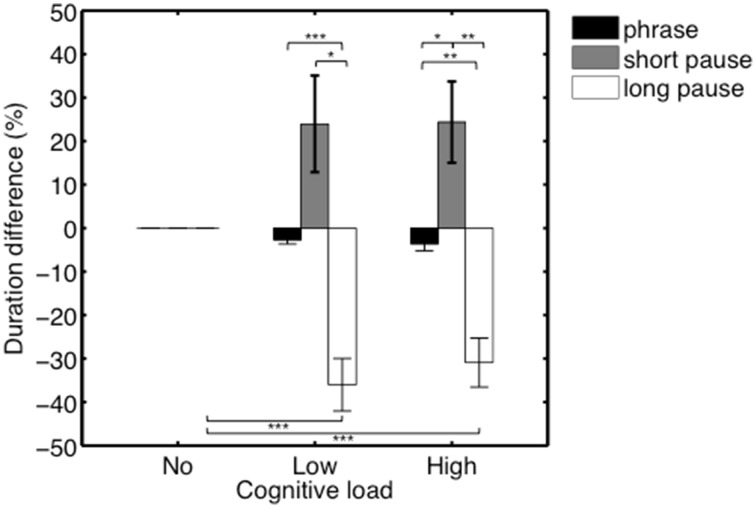
**Average duration difference (in %) of the phrases/short pauses/long pauses in the no/low/high load conditions**. Results show that long pauses are significantly more affected by an additional cognitive load compared to phrases and short pauses. ^*^p ≤ 0.05, ^**^p ≤ 0.01, ^***^p ≤ 0.001.

In order to assess whether effects of heightened cognitive load on the shortening of pause durations were further influenced by the initial duration of the pauses, we conducted an additional analysis. In that analysis, we divided the pauses of each participant into two categories—i.e., long and short pauses—based on a split-median analysis. Categories were made per participant, based on the pauses that occurred in the single Task condition. The medians per participants were respectively 418, 707, 260, 399, 460, 382, 368, 547, 882, 418, 292, 348 ms, (*M* = 456.75 ms, SEM = 51.43). Average duration difference (in %) of the phrases/short pauses/long pauses in the no/low/high load conditions are shown in Figure 5. A Two-Way repeated-measures ANOVA was conducted with Performance type (Phrase/Short pause/Long pause) and Condition (no/low/high) as within-subjects factors. We found a significant main effect of Performance type, *F*_(1.05, 11.55)_ = 18.53, *p* = 0.001, η^2^*_p_* = 0.63. Degrees of freedom were corrected using Greenhouse-Geisser estimates of sphericity, ε = 0.53, as Mauchly's test indicated that the assumption of sphericity had been violated, χ^2^(2) = 23.49, *p* < 0.001. Additionally, we found a significant interaction effect between Performance type and Condition, *F*_(2.05, 22.57)_ = 18.53, *p* = 0.002, η^2^*_p_* = 0.43. Again, degrees of freedom were corrected using Greenhouse-Geisser estimates of sphericity, ε = 0.51, as Mauchly's test indicated that the assumption of sphericity had been violated, χ^2^(9) = 44.52, *p* < 0.001. Results of the *post-hoc* tests can be found in Table 2.

**Table 2:** **Results of the *post-hoc* comparisons of the repeated-measures ANOVA (Performance type, Phrase/Short pause/Long pause; Condition, no/low/high), adjusted for multiple comparisons using Bonferroni's method (SPSS)**.

**PERFORMANCE TYPE**
Phrase (*M* = −2.10, SEM = 0.78) ≠ Short pause (*M* = 16.10, SEM = 5.96) *t*_(11)_ = −2.94, *p* = 0.040
Phrase (*M* = −2.10, SEM = 0.78) ≠ Long pause (*M* = −22.31, SEM = 3.02) *t*_(11)_ = 7.23, *p* = 0.000
Short pause (*M* = 16.10, SEM = 5.96) ≠ Long pause (*M* = −22.31, SEM = 3.02) *t*_(11)_ = 4.48, *p* = 0.003
PERFORMANCE TYPE * TASK
Long pause—No load (*M* = 0, SEM = 0) ≠ Low load (*M* = −36.00, SEM = 6.03) *t*_(11)_ = 5.97, *p* = 0.000
Long pause—No load (*M* = 0, SEM = 0) ≠ High load (*M* = −30.92, SEM = 5.64) *t*_(11)_ = 5.48, *p* = 0.001
Low load—Phrase (*M* = −2.66, SEM = 0.99) ≠ Long pause (*M* = −36, SEM = 6.03) *t*_(11)_ = 5.48, *p* = 0.001
Low load—Short pause (*M* = 23.96, SEM = 11.10) ≠ Long pause (*M* = −36, SEM = 6.03) *t*_(11)_ = 3.68, *p* = 0.011
High load—Phrase (*M* = −3.64, SEM = 1.59) ≠ Short pause (*M* = 24.35, SEM = 9.34) *t*_(11)_ = −2.91, *p* = 0.04
High load—Phrase (*M* = −3.64, SEM = 1.59) ≠ Long pause (*M* = −30.92, SEM = 5.64) *t*_(11)_ = 5.02, *p* = 0.001
High load—Short pause (*M* = 24.35, SEM = 9.34) ≠ Long pause (*M* = −30.92, SEM = 5.64) *t*_(11)_ = 3.84, *p* = 0.008

The interaction effect between Performance type and Condition was mainly driven by a significant decrease of the duration (in %) of long pauses in the low (*M* = −36.00, SEM = 6.03) and high load conditions (*M* = −30.92, SEM = 5.64), compared to the duration (in %) of phrases and short pauses. In contrast, in the high load condition, there was an increase (in %) in the duration of short pauses (*M* = 24.35, SEM = 9.34), relative to the duration of phrases (*M* = −3.64, SEM = 1.59).

As a final step, we wanted to investigate how individual performances related to the general pattern displayed in Figure 5. As observed in Figure 7, that plots individual duration differences across Conditions (no/low/high) per participant, the increase in tempo (i.e., negative percentages) of the longer pauses (plus labels) in the low/high load conditions is generally consistent across the different participants. A further visual inspection indicates that short pauses (diamond labels) in general are becoming longer (positive percentages) in the low/high load conditions. In summary, these observations indicate consistency across participants, reflecting the general pattern displayed in Figure 7.

**Figure 6:**
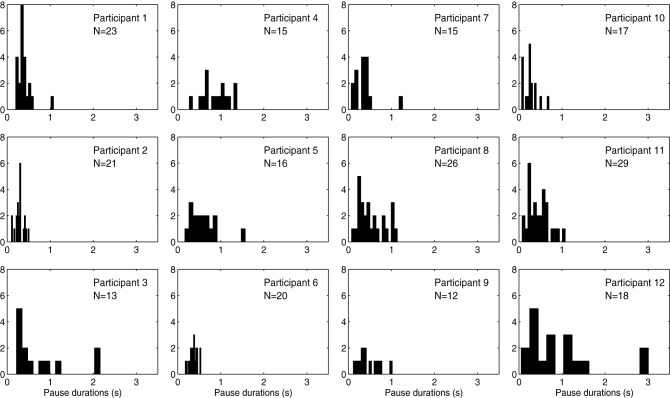
**Histogram shows pauses in no load condition per participant**.

**Figure 7:**
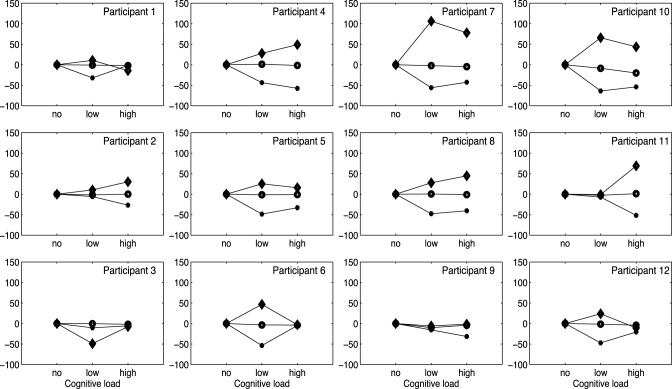
**Graphs show percentage changes of phrase and pauses in three conditions for each participant**. Note that, circles are phrases, diamonds are short pauses, and plusses are long pauses.

## Discussion

The current study positions ongoing cognitive load research into the domain of music performance, more in particular singing performance. Music performance offers us a naturalistic context in which the relationship between timing and cognitive load can be studied. The central question was to what extent a heightened cognitive load affected expressive music performance. For that purpose, we looked at performers' tendency to speed up, or slow down. Previous research demonstrated that timing production generally speeds up in the presence of an additional cognitive load (Krampe et al., 2010; Rattat, 2010; Fischinger, 2011; Çorlu et al., 2014; Maes et al., 2014). Therefore, we expected a general tendency of singers to speed up their performance under cognitive load. Additionally, we made a distinction between the performance of pauses and phrases to test whether an additional cognitive load similarly affects both performance aspects. The results of the study showed indeed that singers have a general tendency to speed up their performance under cognitive load conditions. However, when making a distinction between pauses and phrases, we found that this speeding up was more pronounced in pauses—in particular longer pauses—compared to phrases. This finding suggests that different timing mechanisms underlie the temporal control of pauses and phrases. The fact that the longer pauses are significantly affected by an additional cognitive load as compare to the shorter pauses, suggests the role of a cognitively controlled timing system (Church, 1984; Allman et al., 2014). We expected longer pauses to be more affected by the cognitive load because we expect that there is some more time elapsed apart from respiration. Arguably, during short pauses singer could only breath, which might mean that respiration could function as somatosensory scaffold. The results are in line with our expectations, and as Figure 7 clearly indicates, during longer pauses the devastating effect of an additional cognitive load is significantly higher. When singers breathe they are often trained for example to activate the support and raise the palate to prepare for the next phrase. It can be that less preparation is taken when cognitive load increases. For short pauses, where musicians can only breathe, respiration can serve as a motoric cue to enhance cognitive planning and preparation for the next phrase, becoming part of the atomization process. This might account for the insignificant effect of additional cognitive load in short pauses. Consequently, because of they are shorter and singers were maybe only be able to breathe, therefore motor and somatosensory feedback were still present.

In contrast, the relatively lower impact of an additional load on the timing of phrases suggests that another system supports the timing of phrases. In line with other research, we suggest that the perceptual and motor system—and correspondingly sensory feedback and the control of movement parameters—may directly contribute to temporal control (Jones and Boltz, 1989; Hopson, 2003; Mauk and Buonomano, 2004; Ross and Balasubramaniam, 2014). Through extensive rehearsal and practice, technical aspects of sound production, such as the correct production of singing tones and their durations, become more fluid and ingrained in motoric memory, thus leaving more cognitive resources available for other tasks. Similarly, self-generated auditory feedback, incorporating dynamical patterns of sounds changing over time, may provide temporal cues guiding a singer's performance. The dissociation between a cognitive controlled timing system, and a timing system that is inherently linked to the perceptual-motor system is reflected in the dissociation between *event-based* timing, and *emergent* timing, often mentioned in studies on temporal control of respectively discrete, and continuous rhythmic movements (Robertson et al., 1999; Zelaznik et al., 2002, 2008; Spencer et al., 2003; Huys et al., 2008; Elliott et al., 2009; Lorås et al., 2012; Studenka et al., 2012; Maes et al., 2014). It can be argued that during pause durations—which were made substantially shorter under cognitive load—singers relied on event-based timing. In contrast, research suggests that the temporal control of continuous rhythmic body movements relies on an *emergent* timing system. In that regard, temporal regularities emerge from the motor system's dynamics with a minimum of explicit, cognitive control (Zelaznik et al., 2008). Accordingly, during the singers' performance of musical phrases, continuous activation of phonatory muscles could have functioned as dynamical framework for emergent timing.

For the experiment, we studied professional operatic singers. This category of musicians is especially acquainted with conditions of heightened cognitive load. Their performance does not only involve a musical component, but also an “acting” component in relation to other musicians and an audience. Hence, especially this category of musicians is assumed to develop timing strategies that capitalize on perceptual-motor abilities as an alternative for cognitive resources. Research demonstrated that the specific performance condition of operatic singers (performing under cognitive load, and the assumed development of perceptual-motor timing strategies) is reflected in a specific brain architecture. Kleber et al. (2010) pinpointed specialized neural networks for enhanced somatosensory processing and performance monitoring in operatic singers, as well as motor sequence attention when compared to laymen and even other singers. The findings suggest that changes in the primary S1, IPL, and DLPFC allow for more accurate fine tuning and feed forward motor commands.

An important question is to what extent lyrics could be a factor influencing our results. However, the lyrics were not likely to be the reason for different timing performances simply because they sang the same aria in each condition. Since all musicians shortened the pause durations in load conditions, we rather count on the fact that working memory load is the predominant factor for the differences. From the singers' point of view, singers may change breathing based on the expression of lyrics. However, this would be likely to remain the same between conditions.

The main finding of the present research is that operatic singers speed up their performance, in particular pauses (to be more specific long pauses), in conditions of heightened cognitive load. We expected that this effect would increase proportionally to the level of cognitive load. Therefore, we included two experimental conditions varying only in the level of cognitive load (low/high). However, we found no significant differences between the low load and high load conditions. A possible explanation for this result is that a so-called ceiling effect occurred; the low load condition was already amply difficult in order that an additional cognitive load did not had any further effect on the timing performance.

There are also some limitations that have to be acknowledged. First, the sample size was rather small. It would be of grate necessity that the results presented here, should be confirmed with bigger sample size. Secondly, we are not perfectly sure whether musicians used the shorter pauses solely for breathing. It is plausible to presume that the longer pauses are not only for breathing, but still, to avoid possible uncontrolled variables (such as lung size or capacity) more controlled methodology (using the breath measurer) might be of utmost importance for the validity of our results.

## Conclusion

In this study, we investigated the effect of a heightened cognitive load on the musical performance of operatic singers. Thereby, we focused on musical timing, in particular the tendency of singers to speed up or slow down. Additionally, we differentiated between changes in the duration of musical phrases and pauses. We found that singers, in general, speeded up their performance under heightened cognitive load. Notably, this effect was much more pronounced for pauses, in particular the longer pauses, compared to musical phrases. We suggested that the singers could rely on perceptual and motor resources inherent to the performance of the musical phrases and short pauses to, at least partly, compensate for the disturbing effect of the additional cognitive load task. In contrast during longer pauses singers had to relay on their cognitive resources to keep track of time, which consequently interfered with the performance of the additional cognitive load task. These results provide a better view on the cognitive, sensory, and sensorimotor mechanisms underlying musical timing, and may endow strategies to counteract the disturbing effect of heightened cognitive load on musical performance.

### Conflict of interest statement

The authors declare that the research was conducted in the absence of any commercial or financial relationships that could be construed as a potential conflict of interest.
